# First report on degradation of *Alternaria* toxins in apple juice by UV-C treatment

**DOI:** 10.3389/fnut.2025.1753326

**Published:** 2026-01-13

**Authors:** Ufuk Gokce Ayranci, Hakan Karaca

**Affiliations:** Department of Food Engineering, Faculty of Engineering, Pamukkale University, Denizli, Türkiye

**Keywords:** *Alternaria* toxins, apple juice, color, degradation, UV-C

## Abstract

Toxic metabolites of *Alternaria* spp. are considered as “emerging mycotoxins” and have not been yet regulated by legislation in many countries. *Alternaria* species can grow and produce these mycotoxins in various products including fruits and fruit juices. In the present study, UV-C treatment (3971.09 J m^−2^, 1–8 h) was used for the first time to degrade *Alternaria* toxins [tenuazonic acid (TeA), alternariol (AOH) and alternariol monomethyl ether (AME)] in apple juice. The effect of the treatment on quality characteristics of apple juice was also evaluated. UV-C treatment was very effective in degrading toxins. AME was the most affected toxin (decreased by half) after a treatment for 1 h. Degradation rates of 90.52, 77.01 and 75.92% were observed in the levels of TeA, AOH and AME, respectively, at the end of treatment for 8 h. Soluble solid content, pH and titratable acidity values did not change significantly after UV-C treatment, however turbidity values increased by 92% at the end of treatment. One of the major effects of UV-C treatment was on the color of the apple juice. Transmittance value at 440 nm increased while a^*^ and b^*^ values decreased significantly (*p* < 0.05) which shows that UV-C had a deleterious effect on color pigments of the juice. Significant decreases were observed also in phenolic content and antioxidant activity of the UV-C treated juice. These results demonstrate that UV-C is quite effective in degrading *Alternaria* toxins in apple juice but has detrimental effects on some quality characteristics, especially for prolonged treatment periods.

## 1 Introduction

*Alternaria* is an important fungal genus which is commonly found in many regions of the world (1). Some species of this genus can cause severe diseases, yield reductions and economic losses in various crops (2). For instance, *A. alternata* is responsible for two different diseases in apples namely *Alternaria* blotch which appears as brown spots on the fruit and moldy core characterized by internal decay of the fruit with no external symptoms (3). *Alternaria* infection typically develops under moderate temperatures (25–31 °C), extended leaf wetness and rainfall, which collectively promote spore dispersal and initial infection. Effective preventive measures include the removal of fallen leaves, the timely application of protective fungicides and early-season cultural practices such as pruning or copper-based treatments (4). Although *Alternaria* is known as a “field fungus” as its infection generally occurs before harvest, it can cause severe damage to many crops also during storage and even in cold chain transportation and distribution (5). Moreover, some species of *Alternaria* can produce toxic secondary metabolites (also known as mycotoxins) under favorable conditions such as moderate temperature and high humidity (6). Among these mycotoxins, alternariol (AOH) and alternariol monomethyl ether (AME) have been reported to exhibit pronounced genotoxic, carcinogenic, cytotoxic, and mutagenic activities (7). Moreover, several *Alternaria* metabolites such as AOH, AME, tenuazonic acid (TeA) and altertoxins (ATX) have also been described to induce adverse effects in animals, including fetotoxic and teratogenic outcomes (8).

The number of notifications on *Alternaria* toxins received by the Rapid Alert System for Food and Feed (RASFF) of the European Union increased greatly in the last 5 years. These notifications revealed high levels of TeA contamination up to 15,102 μg kg^−1^ in paprika powder (9) and 5,000 μg kg^−1^ in hazelnuts (10). Currently, the allowed amounts of *Alternaria* toxins in any kind of food have not been restricted by legislation worldwide with the exception of a limit—set by the Bavarian Health and Food Safety Authority—of 500 μg kg^−1^ TeA in sorghum/millet-based infant food (11). *Alternaria* toxins have gained increasing scientific attention following the 2011 report published by the European Food Safety Authority (EFSA) on their occurrence in food and feed (8). In 2016, EFSA emphasized the need for additional data on key *Alternaria* metabolites, including TeA, AOH, AME, altenuene (ALT) and tentoxin (TEN) to enable a more robust risk assessment, and subsequently issued a call for data to better characterize human exposure (12). More recently, in 2022, EFSA introduced indicative levels for AOH, AME and TeA in certain foods, based on the available data in the EFSA database. The food products found in the EFSA list are processed tomato products, paprika powder, sesame seeds, sunflower seeds, sunflower oil, tree nuts, dried figs and cereal based foods for infants and young children (13). Despite the reports on *Alternaria* contaminations in apple fruit (3, 14) and the presence of *Alternaria* toxins in apple products (15, 16), apples and their products are not included in the EFSA list.

Fresh fruits can often be infected with molds. If the external symptoms are easily visible on the infected product, it can be discarded through quality control systems in different postharvest stages or rejected by the consumers themselves. In contrast, if the infected product does not show any apparent external symptoms (like in the case of moldy core caused by *Alternaria* spp.) it cannot be removed through control systems and can reach consumer as fresh or in a processed product (17). Removing the part surrounding the infected area on the fruit was shown as an effective way to reduce contamination level of some mycotoxin (i.e., patulin) in juice as the toxin migration through sound tissues is minor (18), however no information is available on emergent toxins, such as those produced by *Alternaria* spp. (17). Low pH values of the juices (i.e., apple juice) are favorable for the survival and growth of molds. Thermal treatments are conventionally used to limit microbiological and enzymatic changes and thus to extend the shelf life of the juices. However, these treatments can lead to undesirable changes in sensory and nutritional characteristics of the product. Also, thermal treatments (~80 °C) were found to be ineffective in degrading mycotoxins like *Alternaria* toxins (19, 20) and patulin (21, 22) in various juice samples. In contrast to thermal processing—which is effective for microbial inactivation but largely unable to remove mycotoxins—UV-C irradiation operates through a fundamentally different mechanism. UV-C irradiation can initiate free radical oxidation and the resulting reactive oxygen species may contribute to toxin reduction by promoting oxidative degradation pathways (23). In addition, UV-C can induce photochemical reactions such as aromatic ring cleavage leading to the structural breakdown of various mycotoxins (24). These mechanisms provide a strong scientific rationale for investigating whether similar degradation pathways may apply to *Alternaria* toxins.

Given the need for alternative processing methods that function at ambient temperature and minimize quality degradation, nonthermal technologies have gained considerable attention. Among them, UV-C treatment stands out due to its microbial effectiveness and low energy consumption (23, 25). The U.S. Food and Drug Administration (FDA) has recognized UV-C as an alternative to traditional thermal pasteurization for the reduction of human pathogens in juices (26). This recognition is primarily based on the demonstrated microbiological efficacy of UV-C treatment in achieving the required 5-log reduction of relevant human pathogens in juices (26). It has also been reported to reduce certain toxic contaminants, such as patulin, for which the FDA has set an upper limit of 50 μg kg^−1^ in fruit juices (27). UV-C part of the UV light spectrum has the highest energy and consequently the strongest antimicrobial potential. This short wavelength radiations (200–280 nm) inactivate many microorganisms by damaging the structure of their DNA (28). Studies conducted with UV-C revealed that this treatment could inactivate not only the vegetative forms of microorganisms but also their spores (25, 29). Moreover, UV-C treatment has demonstrated efficacy in degrading several mycotoxins, including aflatoxins (30), ochratoxin A (31), zearalenone (32), patulin (33), and deoxynivalenol (34). However, very limited information exists regarding the degradation of *Alternaria* toxins by UV-C (23, 35), and to the best of our knowledge, no studies have examined their degradation behavior in apple juice, despite the detection of TeA (15), AOH (16), and AME (36) in this product category.

It should be noted that nonthermal treatments (just like the thermal ones) can affect various characteristics of the treated products to some extent. Indeed, it was reported that UV-C treatment caused significant changes in color (37), nutritional value (38) and volatile compounds (39) of the products. It is obvious that the range of the changes mentioned strongly depends on factors such as the product properties and the treatment conditions (dose and time). Therefore, experimental studies should be conducted to maximize the effect of the treatments on mycotoxins/microorganisms and to minimize the undesirable changes in sensory and nutritional characteristics of the product.

Despite the increasing number of RASFF notifications related to *Alternaria* contamination (9, 10) and the reported presence of TeA, AOH, and AME in apple-derived products (3, 14–16), no study to date has investigated the degradation behavior of *Alternaria* toxins in apple juice under UV-C treatment. Considering that thermal processing is largely ineffective in breaking down these toxins and that there is a growing demand for nonthermal technologies capable of preserving product quality, it becomes a pertinent research question whether UV-C can simultaneously achieve toxin reduction while maintaining the quality attributes of fruit juice. In this context, exploring the potential of UV-C treatment to mitigate *Alternaria* toxins without compromising juice quality is of significant scientific relevance.

The present study hypothesised that UV-C treatment would result in the statistically significant degradation of major *Alternaria* toxins (AOH, AME and TeA) in apple juice, achieving high degradation efficiencies (≥90%) compared to untreated controls. In addition, it was hypothesised that UV-C treatment would not substantially deteriorate the quality of apple juice, with changes to key quality characteristics (such as antioxidant activity and phenolic content) remaining limited to ≤20% compared to control samples. To test these hypotheses, we aimed to determine the effects of different UV-C treatment times on toxin degradation and on several quality attributes of the juice, including soluble solids content, pH, titratable acidity, turbidity, color parameters, total phenolic content, and antioxidant activity. Although no AI tools were applied in the present work, the parametric nature of UV-C processing makes it a promising candidate for future machine-learning-based modeling and optimization studies.

## 2 Materials and methods

Analytical standards of TeA (>90%) and AOH (94%) were purchased from Toronto Research Chemicals (Ontario, Canada). Analytical standard of AME (99%) was obtained from Santa Cruz Biotechnology Inc. (Heidelberg, Germany). AOH and AME standards were stored at −18 °C while TeA standard was stored at −80 °C. Apple juice concentrate (65° Brix, clarity at 625 nm, Transmittans (T)% <90, color at 625 nm, T% <70–80, turbidity <1 NTU, pH 3.2–4.0) was kindly supplied by a commercial juice manufacturing company (Anadolu Etap, Denizli, Türkiye). The concentrate was diluted with deionized water at room temperature to 11.2° Brix. All juice samples were prepared under aseptic conditions. A total of 2,600 mL of sample was prepared; half of this amount was contaminated with *Alternaria* toxins and used in degradation studies (as described below) and the other half was used to determine the effect of the treatment on physical and chemical properties of the juice. A portion of 300 mL of toxin-free apple juice that was not treated with UV-C was served as the negative control. All diluted samples were kept at +4 °C until use. After UV-C treatment, samples intended for toxin quantification were analyzed immediately, whereas aliquots reserved for quality analyses were stored at −18 °C until analysis.

### 2.1 Preparation of contaminated juice samples

Since the prices of *Alternaria* toxin standards are quite high, the toxins used to prepare contaminated apple juice were extracted from microbiological medium on which toxigenic *A. alternata* grew instead of spiking toxins directly to the juice. For this aim, an *A. alternata* strain (American Type Culture Collection-ATCC 34957, isolated from sorghum hybrid “Pioneer 8442”) which is known to produce TeA, AOH and AME toxins (40) was used. Before being used in the experiments, the strain was activated on potato dextrose agar (PDA) at 30 °C for 10 days. After activation, spores were harvested by adding 10 mL of sterile distilled water and scraping the surface of the medium with a Drigalski loop. The obtained suspension was filtered through a cheesecloth and the density of the spores in the suspension was measured with a hemocytometer. The required dilutions were made with sterile distilled water to obtain an inoculum density of 5.3 × 10^6^ spores mL^−1^. A total of 40 petri dishes containing PDA were prepared and each dish center was inoculated with 10 μL of the spore suspension prepared. The inoculated dishes were incubated at 30 °C for 10 days. After this incubation period, the surface of the medium was completely covered with fungal mycelia and toxins were extracted from this medium.

For the extraction of *Alternaria* toxins, PDA media on which the fungus had grown for 10 days were transferred from 40 petri dishes to a Waring blender and blended for 1 min at high speed. Then, 200 mL of methanol was added to the blender and blended again for 1 min. The contents of the blender were transferred to an Erlenmeyer flask and this flask was shaken for 30 min on an orbital shaker (OS-20, Boeco, Hamburg, Germany). This mixture was filtered through a coarse filter paper and the resulting filtrate was refiltered through Whatman No. 1 (Cytiva, Little Chalfont, Buckinghamshire, United Kingdom) into a rotary evaporator flask. The filtrate in the flask was evaporated in a rotary evaporator (Scilogex RE 100-PRO, CT, United States) at 50 °C to a volume of ~30 mL. This methanolic extract served as the toxin stock solution. A defined volume of this extract was then mixed with 1,300 mL of apple juice (11.2° Brix) to obtain initial toxin concentrations of approximately 791.50 μg L^−1^ for TeA, 71.37 μg L^−1^ for AOH, and 34.39 μg L^−1^ for AME in the contaminated juice. Before UV-C treatment, toxin concentrations in the contaminated apple juice were quantified by HPLC and toxin homogeneity was confirmed by analyzing samples taken from different depths. A portion of 300 mL of the contaminated apple juice that was not treated with UV-C was served as the positive control. The contaminated juice sample was kept at +4 °C until use.

### 2.2 UV-C treatment

**Figure 1:**
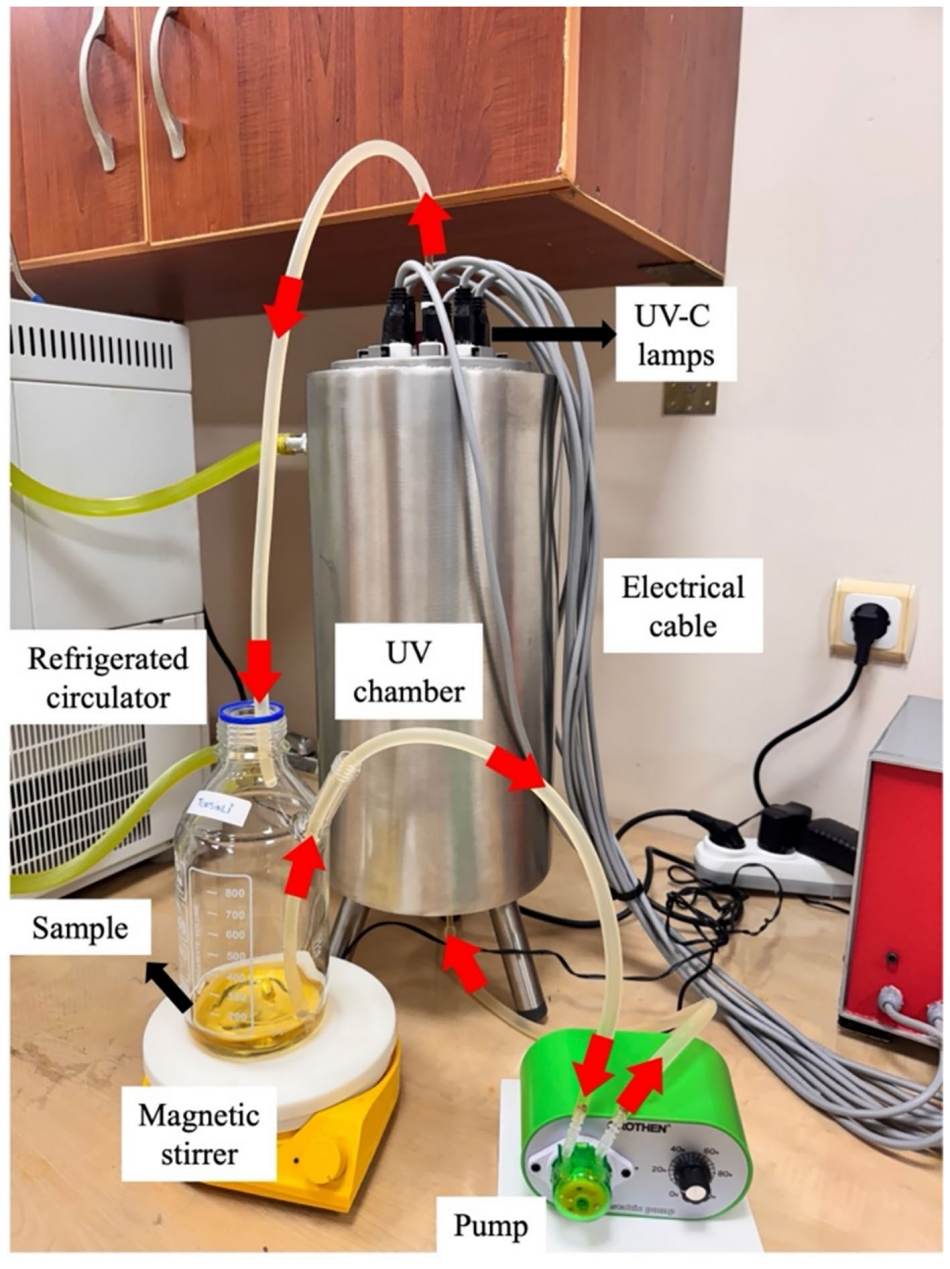
A photo of experimental setup for UV-C treatment.

UV-C treatment of juice samples was carried out in a system consisted of a stainless-steel cylindrical chamber with a wall thickness of 1.5 mm, a flow-speed adjustable peristaltic pump (G328A, Grothen, China) and a magnetic stirrer (MSH-20A, Daihan Scientific, South Korea) to ensure homogeneity of the sample. A cooling liquid at +4 °C obtained from a refrigerated circulator (BD 402, Nuve, Ankara, Türkiye) was circulated through the outer jacket of the chamber to remove the heat coming from the UV-C lamps. By this way, the temperature of the juice circulating through the system could be maintained at 20–22 °C throughout the treatment. Maintaining the juice at 20–22 °C prevents any thermal contribution to toxin degradation and avoids temperature-induced biochemical responses that could confound the effects of UV-C. As UV-C exposure and temperature shifts may stimulate oxidative stress pathways and influence phenolic metabolism, controlling the temperature ensures that the reductions observed are due solely to UV-C treatment (41). A photograph of experimental setup for UV-C treatment is given in Figure 1.

**Figure 2:**
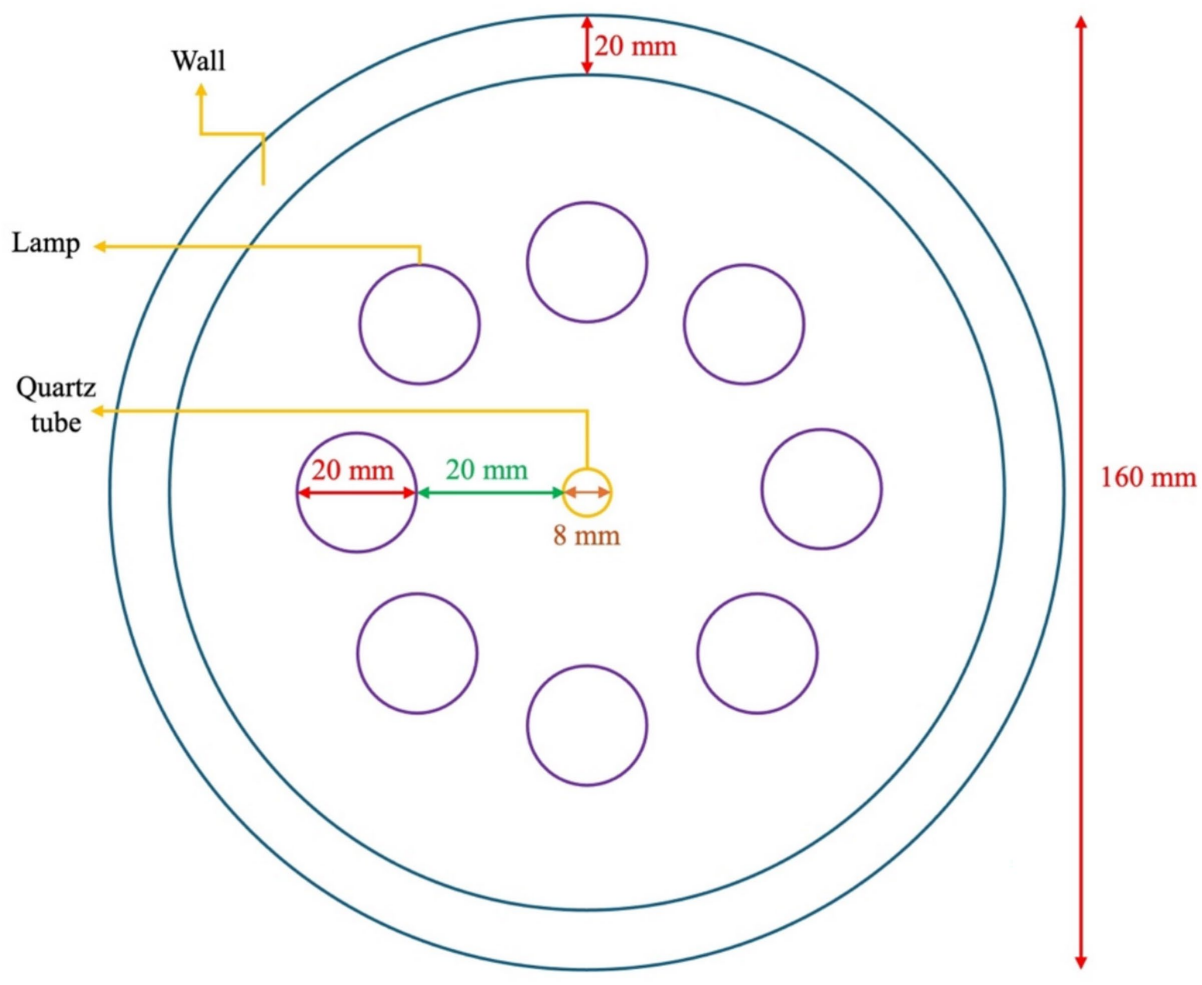
Schematic top view of the UV-C irradiation chamber.

The juice was passed through a quartz tube (height: 37 cm; inside diameter: 5 mm; outside diameter: 6 mm) placed along the axis of the cylindrical chamber at a flow rate of 33 mL min^−1^. Eight UV-C lamps (254 nm, 48 W, 436 mm; LMP-GPH436T5L/HO/4PSE, Atlantic Ultraviolet Co., Hauppauge, NY, United States) were symmetrically positioned around the quartz tube at an equal distance of 2 cm to ensure uniform irradiation along the entire flow path. The central placement of the quartz tube, together with the symmetrical lamp arrangement, provided homogeneous UV-C exposure, while the controlled flow rate and continuous mixing minimized shadowing effects and helped maintain uniform light distribution within the treated juice (Figure 2). This configuration ensured that all portions of the juice were repeatedly exposed to the same UV-C intensity distribution, providing a uniform and reproducible treatment. Juice samples were treated with UV-C light for 1, 2, 4 and 8 h and each treatment time was repeated in triplicates.

UV-C dosage in our system was calculated according to Equation 1 suggested by Keyser et al. (42).


$$\mathrm{Dosage}\;\left(\;J\;m^{-2}\right)=\mathrm{Intensity}\;\left(W\;m^{-2}\right)\times \mathrm{Retention time}\;\left(s\right)$$ (1)

The Retention time in Equation 1 was calculated using Equation 2.


$$\begin{array}{l} \mathrm{Retention time}\;\left(s\right)=\mathrm{Volume of the quartz tube}\;\left(L\right)/\\ \mathrm{Flow rate}\;\left(L\;s^{-1}\right) \end{array}$$ (2)

Here, the flow rate was adjusted using the peristaltic pump and set to 33 mL min^−1^ (0.00055 L s^−1^). The Volume of the quartz tube was calculated using Equation 3.


$$\mathrm{Volume of the quartz tube}\;\left(L\right)=\pi r^{2}\times h$$ (3)

The height of the tube was 37 cm and inner radius was 0.25 cm. So, the volume of the quartz tube was calculated as 7.26 cm^3^ (0.00726 L). Then, the retention time was calculated as 0.00726 L/0.00055 L s^−1^ = 13.2 s. The intensity in Equation 1 was determined using a radiometer (HD 2302, Delta OHM, Italy), the probe of which was placed at the same position as that of quartz tube. Three replicate measurements were taken from three different positions using the radiometer. The intensity was measured as 37.61 W m^−2^ for one single lamp and calculated as 300.84 W m^−2^ for 8 lamps in total. Then, the dosage was calculated as 300.84 W m^−2^ × 13.2 s = 3971.09 J m^−2^.

### 2.3 Analysis of *Alternaria* toxins

Analysis of *Alternaria* toxins in treated and untreated apple juice samples was carried out according to the method of da Motta and Valente Soares (43) with a slight modification. Accordingly, 10 mL of apple juice sample and 40 mL of methanol were mixed and this mixture was stirred for 2 min in a magnetic stirrer. After adding 12 mL of ammonium sulfate solution (10%, w/v), the mixture was stirred for an additional 10 min and filtered through coarse filter paper. The filtrate was transferred into a separating funnel and 10 mL of distilled water at 8 °C was added. The mixture in the separating funnel was extracted with 4 mL of chloroform for 2 min. This extraction step was repeated one more time using 4 mL of fresh chloroform. The chloroform extracts-which contain AOH and AME-were combined in a beaker. The remaining phase in the separating funnel was transferred to an Erlenmeyer flask and its pH was adjusted to 2 with 6 N HCl. This solution was extracted two times with chloroform (2 × 4 mL) as explained above. The resulting extract—which contains TeA—was combined with the previously obtained extracts. The combined extracts were filtered over anhydrous sodium sulfate and the filtrate was evaporated to dryness in the rotary evaporator at 40 °C. The residue was dissolved in 4 mL of high performance liquid chromatography (HPLC) grade methanol and the resulting sample was filtered through a 0.22 μm filter.

HPLC analysis was carried out using a Thermo Scientific Dionex Ultimate 3000 chromatograph equipped with a pump (LPG-3400SD), a column compartment (TCC-3000SD) and a diode array detector (DAD-3000) set at 258 nm for AOH and AME, and at 280 nm for TeA determination. The analytical column was Inertsil ODS 3 (GL Sciences, 5 μm, 250 × 4.6 mm i.d., Tokyo, Japan), the mobile phase was a mixture of methanol:water (80:20, v/v) containing 300 mg L^−1^ ZnSO₄·H₂O at a flow rate of 0.3 mL min^−1^ and the injection volume was 20 μL.

Stock solutions of AOH, AME and TeA were prepared by dissolving analytical standards of these toxins in HPLC grade methanol (10,000 μg L^−1^, 10,000 μg L^−1^ and 50,000 μg L^−1^, respectively). Standard solutions prepared by appropriate dilutions of these stock solutions were used to construct five-point calibration curves covering the range of 5–250 μg L^−1^ for AOH, 2.5–50 μg L^−1^ for AME and 100–2,500 μg L^−1^ for TeA. Calibration curves with low concentrations of toxins (0.25–2.50, 0.5–2.0 and 1–20 μg L^−1^ for AOH, AME and TeA, respectively) were also drawn to determine limit of detection (LOD) and limit of quantification (LOQ) values. LOD and LOQ values were calculated from these curves by multiplying the standard deviation of the response by 3.3 and 10, respectively, and dividing by the slope of the calibration curve. LOD values were determined as 0.32, 0.14 and 1.31 μg L^−1^ and LOQ values were determined as 0.98, 0.41 and 3.98 μg L^−1^ for AOH, AME and TeA, respectively. Recovery tests were conducted by spiking apple juice samples with the standard solutions of the toxins to obtain known final concentrations. After leaving the samples for 30 min at room temperature, the extraction and the injection procedures were done as explained above. Triple injections were performed and the mean recovery values were calculated as 96, 88 and 120% for AOH, AME and TeA, respectively. All results given in this study were corrected for average recovery values.

### 2.4 Determination of soluble solids, pH, titratable acidity, turbidity and color values

The soluble solids, pH, titratable acidity, turbidity and color values of the apple juice samples were determined before and after the UV-C treatments. Soluble solid content was determined using an Abbe refractometer (Abbe-Ref 2, PCE Instruments, Germany). A pH-meter (Isolab, Eschau, Germany) was used to measure the pH values of the samples. Titratable acidity was determined by titrating with 0.1 N sodium hydroxide solution and expressed as malic acid (g 100 mL^−1^). Turbidity values were determined using a turbidimeter (2100Q, Hach, Loveland, CO, United States) and expressed as nephelometric turbidity unit (NTU). All parameters were evaluated based on the official methods provided by the International Fruit and Vegetable Juice Association (44).

The effects of the treatments on juice color were determined by measuring the transmittance of the samples at 440 nm against a blank (distilled water) using a spectrophotometer (UV-1201V, Shimadzu spectrophotometer, Kyoto, Japan). In addition, colorimetric values ( $L_{0}^{*}$ , $a_{0}^{*}$ and $b_{0}^{*}$ ) of the juice samples were determined using a colorimeter (CSM 1, PCE Instruments, Southampton, United Kingdom). The color difference (∆*E*) between treated and untreated samples was calculated using Equation 4.


$$\Delta E=\sqrt{{\left(L^{*}-L_{0}^{*}\right)}^{2}+{\left(a^{*}-a_{0}^{*}\right)}^{2}+{\left(b^{*}-b_{0}^{*}\right)}^{2}\;}$$ (4)

In the equation, $L_{0}^{*}$ , $a_{0}^{*}$ , $b_{0}^{*}$ were the values of the untreated sample and $L^{*}$ , $a^{*}$ , $b^{*}$ were the values of the treated samples. According to Yamauchi and Kanazawa (45), a classification based on ∆*E* values could be done as follows: larger than 12, another color group; 6.0–12.0, large difference in the same color group; 3.0–6.0, detectable by ordinary people; 1.5–3.0, detectable by trained people; 0.5–1.5, hard to detect with the human eye; 0–0.5, trace difference.

### 2.5 Determination of total phenolic content and antioxidant activity

A gallic acid stock solution was prepared by dissolving 5 mg of gallic acid in 10 mL of distilled water. A series of standard solutions covering a concentration range of 5–100 mg L^−1^ were prepared from this stock solution. A calibration curve (*R*^2^ = 0.9974) was constructed using these standard solutions. The total phenolic contents of the samples were determined using this curve and expressed as mg gallic acid equivalent (GAE) per 100 mL. Total phenolic contents of control and UV-C treated apple juice samples were determined according to the method of Singleton et al. (46). Briefly, 1 mL of 2-fold diluted juice sample and 5 mL of 10-fold diluted Folin–Ciocalteu reagent were put in a test tube and mixed vigorously using a vortex mixer for 1 min. The tube was held in the dark for 1 min and 4 mL of sodium carbonate solution (20%, w/v) were added. After mixing for an additional 1 min and holding at 20 °C for 2 h, the absorbance measurements were taken at 760 nm by the spectrophotometer.

A trolox stock solution was prepared by dissolving 5 mg of trolox in 10 mL of distilled water. A series of standard solutions covering a concentration range of 10–50 μM were prepared from this stock solution. A calibration curve (*R*^2^ = 0.9982) was constructed using these standard solutions. Antioxidant activities of the samples were determined using this curve and expressed as μmol trolox equivalent (TE) per 100 mL. Antioxidant activities of control and UV-C treated juice samples were determined according to the method of Brand-Williams et al. (47). A stock solution of 2,2-diphenyl-1-picrylhydrazyl (DPPH) was prepared by dissolving 24 mg of DPPH in 100 mL of methanol. Then, DPPH working solution was prepared by diluting this stock solution to give an absorbance of about 1.1 at 515 nm. According to the method, 150 μL of 2-fold diluted juice sample and 2,850 μL of DPPH working solution were put in a test tube and mixed vigorously using a vortex mixer for a min. After holding at 20 °C for 1 h in the dark, the absorbance measurements were taken at 515 nm by the spectrophotometer.

### 2.6 Statistical analysis

Statistical analysis of the experimental data was obtained from Minitab 16 Statistical Software (Minitab LLC, State College, PA, United States). One-way analysis of variance (ANOVA) was performed to determine the significance of differences between group means. All experiments were performed in triplicate (*n* = 3) and statistical analyses were conducted using the same number of independent measurements. The differences among the means were tested using Tukey’s multiple comparison test at a significance level of *p* < 0.05.

## 3 Results and discussion

### 3.1 Effect of UV-C treatment on *Alternaria* toxins in apple juice

**Figure 3:**
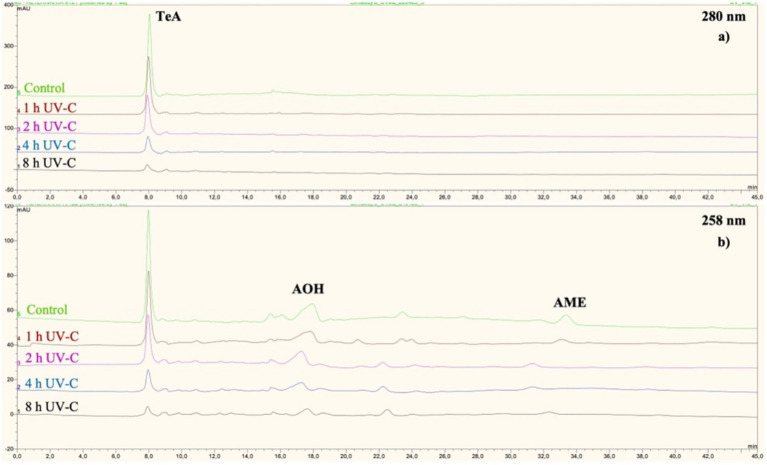
Overlay of chromatograms showing *Alternaria* toxins in apple juice samples treated with UV-C for different time periods: **(a)** 280 nm for TeA **(b)** 258 nm for AOH and AME.

**Figure 4:**
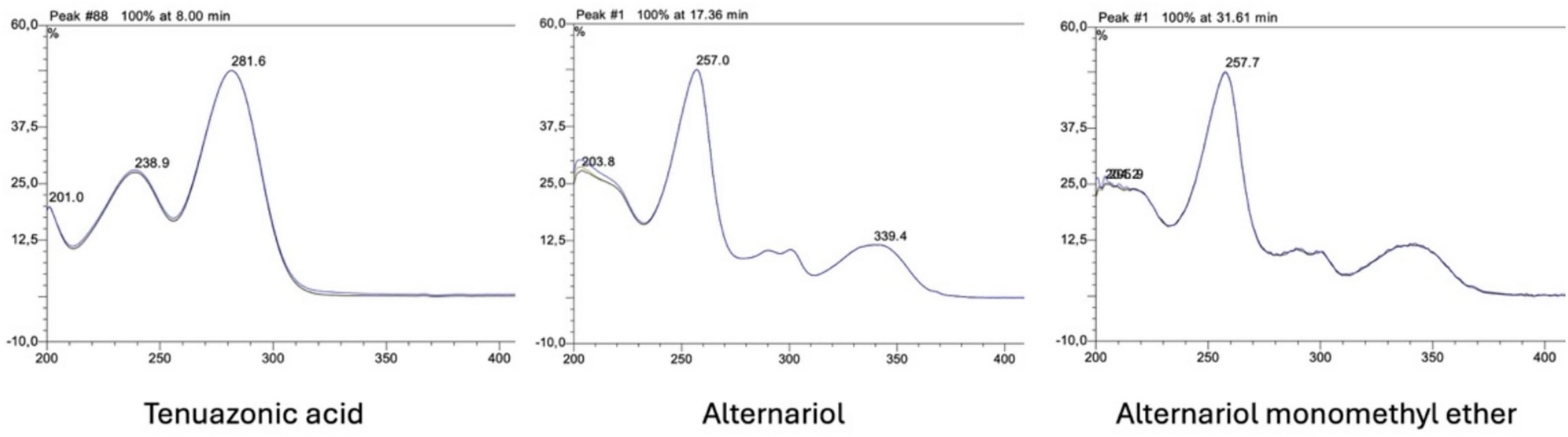
UV spectra of *Alternaria* toxins.

Chromatograms showing peaks of *Alternaria* toxins (for TeA at 280 nm; for AOH and AME at 258 nm) in control and UV-C treated apple juice samples can be seen in Figure 3. Retention times of TeA, AOH and AME were 8.0, 17.4 and 31.6 min, respectively, under the experimental conditions tested. These retention times were in accordance with those of the analytical standards. Moreover, the UV-spectra obtained for the peaks of each analyte were in accordance with the published data of Ashour et al. (48). UV-spectra of the toxins are given as Figure 4.

The concentrations of *Alternaria* toxins in control and UV-C treated apple juice samples are given in Table 1. As can be seen from the table, TeA was the most abundant toxin in control samples, followed by AOH and AME. The concentrations of these toxins in apple juice were 791.50, 71.37 and 34.39 μg L^−1^, respectively. These concentrations are quite rational when the indicative levels introduced by EFSA (12) for these toxins in various food products are taken into consideration (2–30 μg L^−1^, 2–30 μg L^−1^ and 100–10,000 μg L^−1^ for AOH, AME and TeA, respectively).

**Table 1:** Effects of UV-C treatment for different time periods on the *Alternaria* toxins of apple juice^*^.

UV-C treatment		TeA		AOH		AME	
		Concentration (μg L^−1^)	Degradation (%)	Concentration (μg L^−1^)	Degradation (%)	Concentration (μg L^−1^)	Degradation (%)
Treatment time	Control	791.50 ± 93.76^a^	—	71.37 ± 8.41^a^	—	34.39 ± 2.64^a^	—
	1 h	640.17 ± 62.16^b^	19.12	62.16 ± 7.52^ab^	12.89	16.84 ± 1.84^b^	51.02
	2 h	452.34 ± 53.89^c^	42.85	53.89 ± 5.90^b^	24.48	14.30 ± 1.30^bc^	58.41
	4 h	264.86 ± 36.93^d^	66.54	36.93 ± 3.51^c^	48.25	11.20 ± 1.14^cd^	67.44
	8 h	75.00 ± 16.40^e^	90.52	16.40 ± 2.87^d^	77.01	8.28 ± 1.24^d^	75.92

* Data represent mean values and standard deviations (*n* = 3). Differences between means indicated by different letters are statistically significant according to Tukey’s test (*p* < 0.05).

Degradation of all *Alternaria* toxins tested in the present study increased significantly with increasing the UV-C treatment time (*p* < 0.05). It might be due to the enhanced formation of reactive oxygen species (ROS)—which are capable of degrading toxins—as the UV-C treatment time prolonged. A similar situation was also reported by Udovicki et al. (24) who studied the effect of UV-C light on aflatoxin B1. In addition to ROS accumulation, prolonged UV-C exposure provides more opportunities for hydroxyl radicals (•OH) to attack the aromatic rings and phenolic hydroxyl groups of *Alternaria* toxins, initiating oxidative cleavage and structural destabilization. According to photochemical principles and earlier research, UV-C photons possess sufficient energy to break O–H, C–C, and C–O bonds, and this cumulative bond-scission effect becomes more pronounced as the irradiation time increases (23). Therefore, both the increased radical density and the extended duration of molecular photolysis likely contribute to the higher degradation rates observed at longer treatment times.

The shortest treatment time tested in the present study (1 h) resulted in 19.12, 12.89 and 51.02% degradation in the levels of TeA, AOH and AME, respectively. It shows that after 1 h treatment, AME was the most affected one among the tested toxins. On the contrary, Han et al. (23) reported that AOH was more easily degraded than AME after a 90-min UV-C treatment in model system. According to the authors, there are three hydroxyl groups of AOH, which caused it to be more easily oxidized than AME that possesses only two hydroxyl groups on its benzene ring. The same authors reported that citric and malic acid, abundantly found in our samples (apple juice) (49), could increase the degradation of both AME and AOH (23).

Degradation of all *Alternaria* toxins continued throughout the treatment period tested. Towards the end of the treatment, degradation rate of TeA and AOH increased compared to the beginning. For example, degradation rates recorded for TeA were 19.2 and 42.85% at the end of 1 h- and 2 h-treatments, respectively. Also, AOH was degraded by 24.48, 48.25 and 77.01% with UV-C treatment for 2, 4 and 8 h, respectively. It is obvious that UV-C affects not only the toxins but also the other compounds like phenolics, vitamins in the apple juice (50). At the beginning of the treatment, these compounds (the ones other than toxins) might absorb a substantial amount of UV-C (51). Later on, their structure might change, and their UV-C absorption might be reduced. This can be a logical explanation for the enhanced degradation of the two most abundant toxins in our samples.

In the literature, there are very few studies investigating the effect of UV-C treatment on *Alternaria* toxins. In one of these studies, UV-C light was used to reduce the amount of toxins produced by *A. alternata* and to inhibit their penetration into tomato fruit. The authors found that both toxin production and penetration could be effectively reduced by UV-C treatment (35). In another study, the effect of UV-C light on degradation of *Alternaria* toxins was investigated. After UV-C treatment for 90 min, AOH in methanol, aqueous solution and solid state was degraded by 89.1, 72.9 and 53.2%, respectively, while the degradation percentages of AME were 86.6, 50.1 and 11.1%, respectively (24). It can be clearly seen from these results that the effectiveness of UV-C light strongly depends on the medium treated. The penetration of the UV-C light is higher in liquids than in solids. Our sample (juice) is similar to the aqueous solution among the media tested in the aforementioned study. The degradation rates of AOH and AME reported by the authors for this medium (50.1–72.9%) are quite close to those obtained in the present study.

It was shown that *Alternaria* toxins can transfer from the infected fruits into the juice if these fruits are used as raw material (52). Heat treatment is the most common technique used in the juice industry to inactivate microorganisms and degrade toxins in the juices. However, scientific studies revealed that pasteurization (95 °C) and even heating over 100 °C were not sufficient to reduce some *Alternaria* toxins in various juice samples such as tomato juice (53) and pomegranate juice (19). Therefore, nonthermal treatments were also tested for degrading *Alternaria* toxins in juice and juice-like media. For example, Qi et al. (54) evaluated the efficiency of pulsed light on the degradation of AOH and AME in apple juice. The maximum degradation rates obtained for AOH and AME were 79.2 and 68.7%, respectively. The authors also determined that the degradation rates of the toxins were significantly increased by decreasing the depth of the juice and the distance during the pulsed light treatment. Wang et al. (55) investigated the effectiveness of ozone treatment in degrading AOH and AME in aqueous solution and orange juice. The authors reported that the degradation percentages of AOH and AME in the juice after ozone treatment were 51.86 and 49.04%, respectively, which were comparatively lower than those observed in aqueous solution. In the present study, we tested another nonthermal treatment for degrading *Alternaria* toxins in apple juice. The maximum degradation rates were observed after the longest treatment time tested (8 h). These rates were recorded as 90.52, 77.01 and 75.92% for TeA, AOH and AME, respectively. These degradation rates are very close to (and even higher than) the ones obtained by the other techniques reported in the literature (54, 55). Due to these promising results, UV-C treatment can be a potential alternative to thermal and nonthermal treatments in order to effectively degrade *Alternaria* toxins in apple juice. In contrast to conventional thermal pasteurization, which has been reported to be largely ineffective for the degradation of *Alternaria* toxins (19, 53), UV-C irradiation offers a nonthermal, energy-efficient, and more effective approach capable of achieving substantially higher reduction rates. Although a direct experimental comparison with conventional thermal pasteurization was beyond the scope of the present study, a conceptual comparison can be made based on existing literature. Thermal treatments are well established for microbial safety in fruit juices; however, they are generally ineffective in degrading *Alternaria* toxins and are often associated with adverse effects on heat-sensitive quality attributes such as color, aroma, and nutritional compounds. In contrast, UV-C treatment offers a nonthermal alternative that has shown potential for toxin degradation while operating at lower energy inputs, although its industrial feasibility and cost-effectiveness depend strongly on juice optical properties and reactor design. Besides, before using UV-C treatment in practical applications, its potential effects on quality characteristics of apple juice should be revealed.

### 3.2 Effects of UV-C treatments on quality characteristics of apple juice

The effect of UV-C treatment on soluble solid content, pH, titratable acidity, turbidity and color values of apple juice is given in Table 2. Soluble solid content, pH and titratable acidity values did not change significantly after the UV-C treatments (*p* > 0.05). It shows that the UV-C light does not have a substantial impact on sugars and acids, which are the main contributors to the soluble solids of the apple juice. Similar results were reported for other kinds of juices (56–58). On the other hand, turbidity values of the samples increased as the UV-C treatment time increased. Turbidity value of the control sample was 0.76 NTU whereas the turbidity values of the samples treated with UV-C for 1, 2, 4 and 8 h were 0.88, 0.93, 1.27 and 1.46 NTU, respectively. The turbidity of apple juice is primarily attributed to pectin, proteins, free amino acids, phenolic compounds, and the presence of living or dead microorganisms (56). While thermal treatments are known to disrupt cell wall structures and release pectin, proteins, and other macromolecules into the juice—thereby increasing turbidity—previous studies have not reported a comparable effect for UV-C irradiation. In fact, UV-C has been described as capable of reducing turbidity and improving the visual clarity of apple juice (56). Nevertheless, turbidity responses to UV-C exposure may vary depending on juice composition and matrix complexity. In beverages that are initially low in turbidity, haze formation can occur through protein–polyphenol interactions, leading to the formation of colloidal aggregates (59). Considering these mechanisms, the increase in turbidity observed in the present study may be associated with UV-C-induced alterations in phenolic compounds, partial destabilization of pectin-protein structures, or the formation and dispersion of fine suspended particles within the apple juice matrix.

**Table 2:** Effects of UV-C treatment for different time periods on the soluble solids, pH, titratable acidity, turbidity and color values of apple juice^*^.

Characteristics			UV-C treatment time			
		Control	1 h	2 h	4 h	8 h
Soluble solids (° Brix)		11.2 ± 0.0^a^	11.2 ± 0.0^a^	11.2 ± 0.0^a^	11.2 ± 0.0^a^	11.2 ± 0.0^a^
pH		3.25 ± 0.08^a^	3.26 ± 0.07^a^	3.26 ± 0.05^a^	3.31 ± 0.04^a^	3.29 ± 0.09^a^
Titratable acidity (g 100 mL^−1^)		0.31 ± 0.0^a^	0.31 ± 0.0^a^	0.31 ± 0.0^a^	0.31 ± 0.0^a^	0.31 ± 0.0^a^
Turbidity (NTU)		0.76 ± 0.03^a^	0.88 ± 0.09^ab^	0.93 ± 0.06^bc^	1.27 ± 0.24^c^	1.46 ± 0.15^c^
Color	T% (440 nm)	48.00 ± 0.4^a^	57.80 ± 1.3^b^	60.70 ± 0.8^c^	67.40 ± 2.0^c^	83.30 ± 2.7^d^
	*L* ^*^	47.28 ± 0.45^a^	49.45 ± 0.36^a^	48.43 ± 2.20^a^	47.44 ± 4.22^a^	48.47 ± 2.76^a^
	*a* ^*^	5.11 ± 0.91^a^	3.49 ± 0.57^b^	3.62 ± 0.26^b^	3.03 ± 0.68^bc^	2.48 ± 0.82^c^
	*b* ^*^	21.91 ± 0.81^a^	19.37 ± 1.15^b^	18.81 ± 2.66^b^	14.69 ± 1.17^c^	9.74 ± 0.37^d^
	∆*E*	—	4.11 ± 1.02^a^	4.72 ± 1.17^b^	8.46 ± 2.26^c^	12.78 ± 1.02^c^

* Data represent mean values and standard deviations (*n* = 3). Differences between means indicated by different letters are statistically significant according to Tukey’s test (*p* < 0.05).

Color is one of the most important attributes that affect the consumers’ preference for the juices, as well as other food products. Table 2 shows the effect of UV-C treatment on various color parameters of the apple juice. The transmittance value of the apple juice sample significantly increased as the UV-C treatment time increased (*p* < 0.05). It means that the UV-C treated samples are lighter in color compared to control samples. However, this effect was not correlated with L^*^ values (an indicator of luminosity) which did not change significantly due to UV-C treatment. On the other hand, a^*^ and b^*^ values significantly decreased as the UV-C treatment time increased (*p* < 0.05). Decreases in a^*^ and b^*^ values mean decreases in redness and yellowness, respectively, which is probably due to photodestruction of the color pigments of the juice. These pigments can either be originally present in the juice or can be formed later by the rapid action of polyphenol oxidase (enzymatic browning reactions) as well as the Maillard reaction between sugars and amino acids (58). Our results are in accordance with the ones of Guerrero-Beltrán and Barbosa-Cánovas (60) who reported a simultaneous decrease in a^*^ and b^*^ values of UV-C treated apple juice. They observed that the transparent yellow-brown color of apple juice turned slightly to less red and to less yellow in UV-C treated samples. Decreases in a^*^ and b^*^ values in the juices of other fruits such as peach and lemon as a result of UV-C treatment were also reported (61).

**Figure 5:**
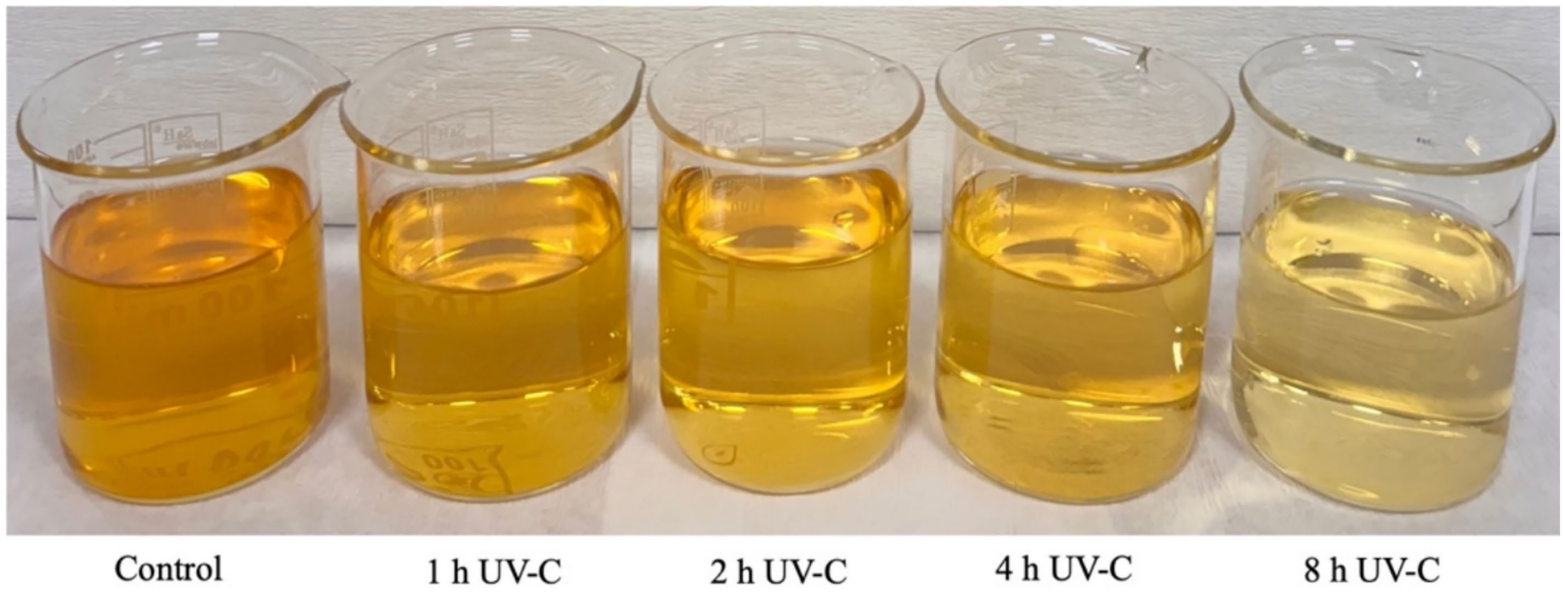
Discoloration of apple juice due to UV-C treatment.

We also calculated the ∆*E* values to evaluate the color differences between the control and UV-C treated samples. These values significantly increased by increasing the UV-C treatment time. For example, ∆*E* values were 4.11 and 4.72 after UV-C treatments for 1 h and 2 h, respectively, which means that the difference observed is “appreciable; detectable by ordinary people” according to the Handbook of Color Science (45). After treatments for 4 h and 8 h, ∆E values were 8.46 and 12.78, respectively. These values correspond to differences that can be classified as “large difference in the same color group” and “another color group,” respectively. Discoloration of apple juice due to UV-C treatment is clearly seen in Figure 5.

**Figure 6:**
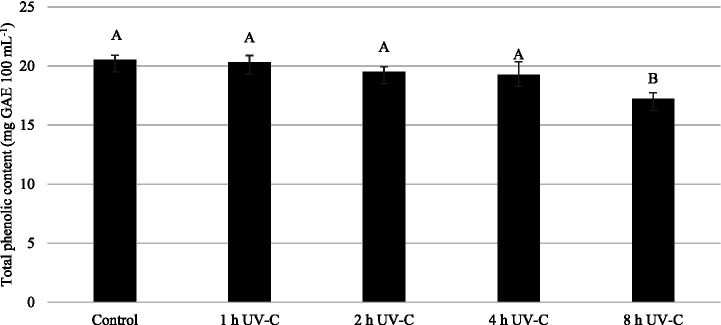
Effect of UV-C treatment for different time periods on the total phenolic content of apple juice^*^. Asterisks indicate data represent mean values and standard deviations (*n* = 3). Differences between means indicated by different letters are statistically significant according to Tukey’s test (*p* < 0.05).

The concentration and composition of phenolics in apple juice are not constant and affected by the raw material and the technology used in production (62). But in general, apples and their products are rich in phenolic acids such as chlorogenic acid, gallic acid, caffeic acid, coumaric acid and flavonoids such as catechin, phloridzin, quercetin, quercetin glycosides (63). The effect of UV-C treatments for different time periods on the total phenolic content of apple juice is shown in Figure 6. As can be seen from the figure, the total phenolic content did not change during the first 4 h of UV-C treatment and was not significantly different from the control (*p* > 0.05). However, a clear decrease was observed only after 8 h of UV-C exposure (*p* < 0.05), with the 8-h treatment resulting in a 16% reduction in the total phenolic content of the juice. Our results are in parallel with those of Xiang et al. (63), who reported decreases of 3.03–10.45% in the total phenolic content of apple juice following exposure to UV-C LEDs at doses of 200–1,200 mJ cm^−2^. Similar reductions were also noted by Noci et al. (64), who observed a statistically significant decrease in total phenolics after UV-C treatment. In contrast, Islam et al. (38) reported that the total phenolic content of apple juice remained unchanged regardless of UV-C exposure. Additionally, Barut Gök (37) found that UV-C treatment significantly increased the total phenolic content of apple juice, further highlighting the variability in phenolic responses to UV-C depending on the juice matrix and processing conditions.

**Figure 7:**
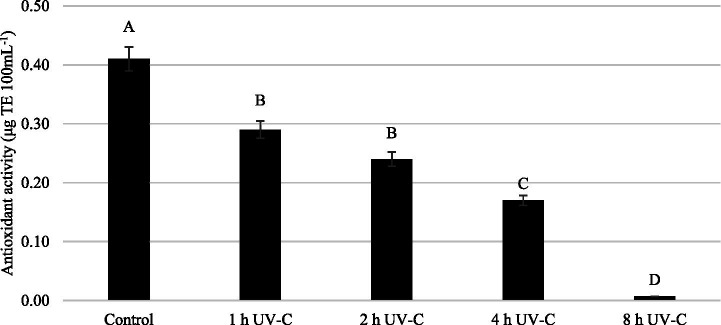
Effect of UV-C treatment for different time periods on the antioxidant activity of apple juice^*^. Asterisks indicate data represent mean values and standard deviations (*n* = 3). Differences between means indicated by different letters are statistically significant according to Tukey’s test (*p* < 0.05).

The effect of UV-C treatments for different time periods on the antioxidant activity of apple juice is shown in Figure 7. It is seen from the figure that the antioxidant activity gradually decreased as the treatment time increased. The antioxidant activity of apple juice sample treated with UV-C for 8 h was about only 1.7% of that of the control. In the literature, it was indicated that the antioxidant activity of apple juice is mostly due to phenolic compounds and in particular polyphenolic acids, flavonoids and proanthocyanidins (65). However, our findings on the decrease in antioxidant activity cannot be explained by the decrease in phenolic content since total phenolic content values of our samples maintained for the first 4 h of the treatment. Antioxidant compounds other than phenolics such as chlorophylls and some antioxidant vitamins (vitamins A, C and E) found in apple juice might play a role in the decrease of antioxidant activity observed. Barut Gök (37) reported that fruit juices contain different antioxidant compounds that could react in different mechanisms when treated with UV-C. In accordance with our results, some authors reported decreases in antioxidant activity of UV-C treated apple juice (38, 39, 56). However, it was also reported that antioxidant activity of apple juice did not change (66) and even increased (37) as a result of UV-C treatment. According to Alothman et al. (67), the behavior characteristics of antioxidants in plants and plant-based products differ greatly and are not fully enlightened yet. The effects of ongoing chemical reactions such as enzymatic and non-enzymatic browning reactions on antioxidant activity of the product and the interactions of UV-C light with all these compounds make the situation more complicated to interpret.

Although UV-C treatment substantially reduced toxin levels, its potential impacts on sensory properties such as flavor and aroma should also be considered, especially given the observed changes in color, turbidity, and phenolic quality. While UV-C exposure yielded 70–90% degradation of *Alternaria* toxins, it remains essential to evaluate whether the residual concentrations fall within regulatory or health-based safety thresholds; however, no specific limits have yet been defined for apple juice. These findings highlight the intrinsic balance between food safety and food quality: higher UV-C doses or prolonged exposure may be necessary to achieve sufficient toxin degradation in opaque matrices such as apple juice, but such conditions can simultaneously intensify quality deterioration, as demonstrated in our experiments. From a technological perspective, strategies such as blending UV-C treated juice with untreated batches may help achieve an acceptable compromise by improving toxin safety while preserving sensory and nutritional attributes. In practical industrial applications, the optimization of UV-C dose and treatment time—aimed at achieving significant toxin reduction with minimal sensory and nutritional losses—should therefore be carefully considered. In order to address the quality deterioration that has been observed at prolonged UV-C treatment times, additional process optimisation approaches may be implemented. These include the treatment with higher UV intensities for shorter exposure times, multiple-pass short-time treatments, or improved reactor designs that enhance UV dose distribution and reduce overall treatment time. Furthermore, the combination of UV-C processing with other non-thermal technologies (e.g., ultrasound, pulsed light) may facilitate synergistic toxin degradation while minimising adverse effects on sensory and nutritional attributes. Preliminary steps, such as clarification or filtration, have been shown to enhance UV penetration in opaque juice matrices. In addition, post-treatment strategies, including antioxidant addition, have been reported to mitigate oxidative quality losses associated with intense UV exposure. Collectively, these integrated approaches underscore the potential of process integration and optimization to surmount the quality challenges associated with protracted UV-C exposure in industrial juice processing.

The findings of this study provide specific insights into the effectiveness of UV-C treatment for reducing *Alternaria* toxins in apple juice and its impact on juice quality, thereby allowing validation of the proposed hypotheses. The first hypothesis was confirmed as UV-C treatment resulted in a statistically significant degradation of the major *Alternaria* toxins (AOH, AME, and TeA), with degradation efficiencies up to 90% at prolonged treatment times in comparison with untreated controls. The reduction of toxins was found to increase in a consistent manner as a function of the duration of UV-C exposure, thereby demonstrating the efficacy of UV-C irradiation in mitigating *Alternaria* toxins in apple juice. With regard to the second hypothesis, which was partially confirmed, UV-C treatment caused limited changes in selected quality characteristics at shorter treatment periods; however, extended UV-C exposure led to pronounced reductions in antioxidant activity, exceeding the predefined 20% threshold.

The limitations of the present study are as follows: The experiments were conducted using artificially spiked apple juice samples containing three *Alternaria* toxins (AOH, AME, and TeA). It should be noted that these toxins may not fully reflect the complexity of naturally contaminated matrices where toxins coexist with fungal spores, secondary metabolites, and native microflora. The study was confined to a single juice matrix and did not include validation against naturally contaminated samples or pathogenic microorganisms. Furthermore, it did not assess potential synergistic or antagonistic interactions among multiple contaminants. Moreover, the shelf-life stability, microbiological safety, and sensory attributes of UV-C-treated juice during storage were not evaluated. Despite the substantial toxin degradation achieved, the relatively long treatment times applied under laboratory conditions give rise to questions regarding industrial feasibility and throughput constraints in commercial juice processing. Additionally, the absence of established regulatory maximum limits for *Alternaria* toxins in apple juice complicates direct compliance assessment. Finally, the chemical identity and toxicological relevance of degradation by-products formed during UV-C treatment were not investigated and remain a critical knowledge gap that must be addressed before industrial implementation.

## 4 Conclusion

In this study, the effectiveness of UV-C treatment in degrading *Alternaria* toxins in apple juice was clearly demonstrated. Degradation increased with treatment time, reaching 90.52, 77.01, and 75.92% for TeA, AOH, and AME, respectively, after 8 h of exposure. However, UV-C treatment simultaneously caused notable changes in key quality attributes of the juice. For example, UV-C treatment for 8 h resulted in more than 50% decreases in redness (a^*^) and yellowness (b^*^) values, an increase over 90% in turbidity and more than a 95% decrease in antioxidant activity of the apple juice. These findings highlight the inherent technological challenge of balancing effective toxin degradation with the preservation of desirable sensory and nutritional properties. To combat these challenges more comprehensive experimental setups should be designed and advanced statistical analyses such as Pearson correlation should be employed to examine the relationships between tested parameters.

It should also be recognized that natural components of the juice, such as pigments and suspended solids, can limit UV-C penetration and thereby reduce treatment efficiency in opaque liquid matrices. However, although UV-C was highly effective in reducing toxin levels, its industrial adoption ultimately depends on confirming the safety of the degradation products that may form during or after treatment. The degradation of *Alternaria* toxins under UV-C irradiation may lead to the formation of unknown transformation products, whose chemical identities and toxicological relevance remain largely unexplored. Given this knowledge gap, the characterization of these degradation products is critically important for studies assessing the safety of UV-C treatment. Therefore, detailed structural characterization and comprehensive toxicological assessment of these by-products are urgently required before UV-C can be recommended as a safe processing alternative. Future research should focus on characterizing the degradation products of *Alternaria* toxins and evaluating their potential toxicities. Furthermore, studies should extend beyond artificially spiked systems to include naturally contaminated apple juice matrices, as well as the presence of fungal spores or co-occurring microorganisms, to better reflect industrial and real-world conditions. Further investigation is warranted into the effects of UV-C treatment on product shelf life, sensory properties, and consumer-relevant quality attributes. Furthermore, the exploration of combined or sequential processing strategies, such as the utilisation of UV-C in conjunction with alternative mild preservation technologies, may facilitate the effective mitigation of toxins while minimising quality deterioration. Collectively, these research directions would provide a more comprehensive assessment of the applicability and safety of UV-C treatment for fruit juice processing.

Finally, UV-C can be considered a parametric nonthermal technology well suited for future AI-based modeling efforts, particularly for optimizing dose–response relationships and predicting quality-safety trade-offs. While the present study does not directly employ artificial intelligence approaches, the generated data provide a valuable basis for such future model development.

## Data Availability

The original contributions presented in the study are included in the article/supplementary material, further inquiries can be directed to the corresponding author.
